# On a Strategy of Advancement of TMS Based Methods for Studying NCC

**DOI:** 10.3389/fpsyg.2018.02026

**Published:** 2018-10-23

**Authors:** Talis Bachmann

**Affiliations:** School of Law, University of Tartu, Tartu, Estonia

**Keywords:** TMS, NCC, ERP, animal model, perception, masking

## Introduction

The well-known advantages of transcranial magnetic stimulation (TMS) in studying the brain mechanisms of mental processes are its noninvasiveness and possibility to study causal effects in addition to the purely correlational ones. Recent years have been marked by numerous advancements in the methods where TMS is used (e.g., Romei et al., 2016). The powerfulness of TMS approaches is enhanced by combining TMS with EEG recording, allowing understand the nature of TMS effects and their relation to consciousness mechanisms better (e.g., Massimini et al., 2012; Sarasso et al., 2015; Vernet et al., 2015; Nieminen et al., 2016). However, there are also numerous shortcomings and underdevelopment in TMS based commonly adopted experimental paradigms. For example, the combined TMS/EEG research mostly focuses on markers of consciousness states and less so on the mechanisms of contents of consciousness of alert subjects. Moreover, we do not know precisely what exactly happens in neurons and brain tissue in the areas directly under the focus of TMS. This is despite some early promising steps in this direction (e.g., Allen et al., 2014; Murphy et al., 2015). The neurobiological artificially produced non-natural effect caused by a sufficiently strong pulse directly targeted at the area of interest quite likely differs from what would be the natural neural state in this area in response to afference arriving via pathways artificially non-perturbed. This suggests use TMS by evoking pathway activity from areas remote from the cortical locus of interest. There is also a technical limitation when we try to stimulate and record the same area. One promising TMS paradigm is relatively free of the two above noted limitations: (i) stimulation of areas remote from the neurons known to be the carriers of the contents of perception (ii) associated with TMS-evoked potential (TEP) measurement of the effects recorded by the electrodes placed far from the target of TMS (but close to the area of content encoding neurons); (iii) all this ought to be combined with the additional methods allowing to interpret these bioelectric signatures in a meaningful way. While scalp-recorded event-related potential (ERP) and TEP generation mechanisms in human brain continue to be difficult to interpret in terms of their underlying neuronal mechanisms, invasive animal studies can inform us better about these mechanims. In what follows an example of this strategy is suggested.

## The gist of the proposed research strategy

Interpretation of TMS-evoked and modal event related potentials in human subjects can be informed by the results from research with animal models where surface potentials are measured and combined with invasive recording techniques allowing to interpret the underlying neuronal level mechanistic processes that give rise to the deflections of the surface recorded potential. Understandably, any TMS study of neural correlates of consciousness (NCC) which is based on animals has a trivial, different type of limitation in addition to the problems of combined stimulation and measurement–we can't have a report from a mouse or rat on what does it feel like to have a particular conscious experience. But the combination of (1) pertinent animal study allowing to put forward specific hypotheses about how the surface recorded bioelectric potential is brought about by mechanisms at the neuronal level with (2) human study with remotely evoked TEPs in a NCC experiment can bring us closer to real NCC. This is trivially evident because a human subject can report his/her phenomenal experience content and level of experience of this content which is a skill unachievable for an animal.

While there is no completed single pre-planned studies based on the above described strategy, let me briefly review two independent studies as examples of what kind of experiments could be combined as parts of a single research project–one with human subjects carried out with remotely produced TEP recording in the context of NCC experiment and the other with animal model for revealing cellular level mechanisms of surface-recorded bioelectrical potential measurement. Thereafter, I will suggest a speculative interpretation of the results of the human experiment as informed by the results of the animal experiment. The main aim of this is to illustrate the principal strategy advisable for future research involving TMS methods and suggest some theoretical directions for hypothesis formulation.

## Review of the experiments

**Figure 1:**
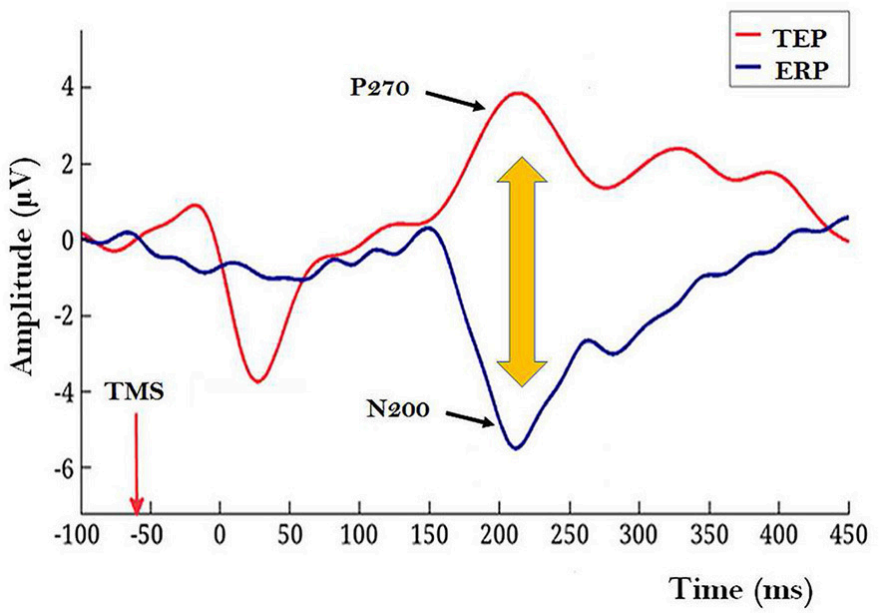
Illustration of a TEP caused by a frontally applied single-pulse TMS (red waveform) and a modal ERP in response to target Landolt stimulus with a conspicuous N200 component (blue waveform). Recordings for these grand averages were made by 27 electrodes located caudally from Cz (10–20 system). In this illustration mutual timing of the ERP and TEP is presented for the condition when TMS is applied 60 ms before the modal Landolt stimulus; this condition allows TEP/P270 overlap with the post-Landolt time epoch where modal ERP N200 is typically observed. (Figure based on data from Rutiku et al., 2016, reused with permission from Elsevier.) Importantly, shorter and longer TMS-to-Landolt SOAs did not produce strong masking.

1. The experiment with human subjects. Rutiku et al. (2016) assessed both objective detection and subjective clarity (4-point scale) of a near-threshold Landolt type stimulus presented timed at −140, −60, or +20 ms SOA with regard to the frontally applied single-pulse TMS. In the studies of TMS-facilitation and TMS-masking of conscious perception TMS is typically targeted at the modal cortical areas implicated in the encoding of the test stimuli (Amassian et al., 1989; Abrahamyan et al., 2015). In Rutiku et al. (2016) the novel TEP-masking method was used. TEP was evoked by *frontal* task-free stimulation and the main region of interest to be tapped by EEG electrodes was located in the *caudal* cortical areas. It was found that the remote-TMS-masking effect on Landolt perception peaked when SOA = −60 ms was used, thus revealing the temporal selectivity of this effect. The modal visual stimulus presentation also produced a typical ERP waveform with a negative peak at about 200 ms post stimulus (see Figure 1, ERP in blue). In many studies on visual NCC the relative amplitude of N200 or VAN (visual awareness negativity) has been shown to correlate with successful conscious perception of the target stimulus (review: Rutiku and Bachmann, 2017) and here this is operationally interpreted as a marker of early visual awareness of the target. The TEP waveform in response to frontal TMS produced a conspicuous positive component P270. Importantly, maximum masking that occurred with SOA = −60 ms took place in the experimental conditions characterized by the temporal coincidence of the TEP P270 and modal ERP marker N200. Neither SOA = −140 nor SOA = +20 produced strong masking. It is inviting to hypothesize that the underlying neural process marked by non-visual TEP/P270 has a strong suppressive effect on the conscious perception specifically when it occurs during neural activity marked by N200 which is a response to modal visual stimulation by the target stimulus. But what does the conspicuous surface-recorded positive potential registered by electrodes posterior from Cz actually mean? For a hypothetical interpretation of it let us now turn to the second study of interest.
2. The experiment capitalizing on an animal model. Suzuki and Larkum (2017) recorded cortical surface potentials in rats in response to optogenetic stimulation of layer-5 pyramidal neurons and to modal sensory stimulation. While surface potentials have been traditionally explained as a result of synaptic events in cortical layers (including in layers deeper than the near-surface layer), Suzuki and Larkum (2017) showed that dendritic calcium spikes occurring in pyramidal neurons are clearly detectable in cortical surface potentials expressed as a conspicuous positive waveform. Related to the NCC domain, it is noteworthy that dendritic Ca2^+^ spikes are more easily evoked in the awake compared to the anesthetized brain (Murayama and Larkum, 2009). Moreover, in a recent theoretical approach to NCC it has been suggested that top-down modulation of tuft apical dendrites and generation of backpropagation activated burst firing of layer-5 neurons may be the mechanism by which specific sensory data are brought in to the contextually modulated contents of perceptual awareness (Larkum, 2013; Phillips et al., 2018).

## Hypothetical synthesis

First, as derived from Suzuki and Larkum (2017) it is postulated here that the task-irrelevant TEP P270 reflects enhanced dendritic calcium activity ignited by the top-down volleys from frontal areas stimulated by TMS. As this process is task-free and can be considered as “dendritic calcium noise” and because conscious perception is hypothesized to be the result of apical amplification from task related top-down context (Phillips et al., 2018), the temporal coincidence of TEP/P270 (the marker of dendritic noise) and N200 (the marker of modal processes necessary for encoding the contents of conscious perception) causes a situation where apical modulation which is characteristic for veridical perception is contaminated. As a result, conscious perception of the target stimulus is suppressed. In order to realize this experimental strategy some interim steps would be needed to validate this approach: Rutiku et al. (2016) design based study must be conducted so as to ascertain the relation between TEP/P270 magnitude and the temporally selective masking effect.

## Concluding remarks

The limitations of this approach at its present stage of development can be easily listed. This in turn presupposes additional studies. First, as dendritic calcium spikes are not the only possible mechanism of the surface-positive potentials, specific studies controlling for the different mechanisms of TEP surface-positivity will be necessary. Second, as the animal model in the Suzuki and Larkum (2017) investigation was applied for somatosensory modality, it remains to be shown that layer-5 visual pyramidal neurons produce similar positive potentials as a result of dendritic calcium activity. Third, there is a need for a special cellular level study to ascertain whether the frontally produced TEP (measured from posterior electrodes) indeed marks the dendritic calcium activity. This can be either an animal study or human study with invasive recordings with neurological patients (or a human MRS study with neurotypical subjects). Yet, despite these issues I believe the scenario depicted in this imaginary *Gedanken*-experimenting can be useful for those who are motivated to follow the strategy of using remotely produced TEPs recorded in the context of human NCC experiments and informed by the animal model studies.

## Author contributions

The author confirms being the sole contributor of this work and has approved it for publication.

### Conflict of interest statement

The author declares that the research was conducted in the absence of any commercial or financial relationships that could be construed as a potential conflict of interest.
